# Effect of a Novel Antidepressant and Anticancer Nuc01 on Depression in Cancer Survivors

**DOI:** 10.3390/cimb47080587

**Published:** 2025-07-24

**Authors:** Changchun Yuan, Xudong Shi, Zhiqiang Wang, Yuqiang Li, Wenbing Ma, Kai Fu

**Affiliations:** 1 School of Chemistry and Chemical Engineering, North University of China, Taiyuan 030051, China; 18735123796@163.com (X.S.); yuqiangli@nuc.edu.cn (Y.L.); mawenbing@nuc.edu.cn (W.M.); kaifu@nuc.edu.cn (K.F.); 2 North University of China Dezhou Industrial Technology Research Institute, Dezhou 253000, China

**Keywords:** PCPA–desvenlafaxine conjugate, LSD1 inhibitor, anticancer, antidepressant

## Abstract

Depression in cancer survivors is commonly treated with serotonin and norepinephrine reuptake inhibitors (SNRIs), such as venlafaxine. These drugs alleviate depressive symptoms by inhibiting the reuptake of serotonin and norepinephrine. However, a novel approach has emerged with the development of *trans*-2-phenylcyclopropylamine (PCPA)–drug conjugates that inhibit lysine-specific demethylase 1 (LSD1), which is a biomarker and molecular target for cancer therapy. LSD1 inhibition can effectively suppress cancer cell proliferation. Nuc01 is a novel PCPA–drug conjugate designed as a prodrug of venlafaxine. In vivo studies showed that Nuc01 dose-dependently reduced immobility time in the tail suspension test in mice, outperforming desmethylvenlafaxine. This suggests that Nuc01 may act as a potent triple reuptake inhibitor, potentially offering enhanced efficacy in the treatment of depression. Additionally, in vitro studies demonstrated that Nuc01 effectively occupies the PCPA binding site within LSD1 (IC_50_ = 530 nm) and inhibits the proliferation of MDA-MB-231 cancer cells (IC_50_ = 1130 nm). These findings suggest that Nuc01 may function as an LSD1 inhibitor with potential anticancer properties. Collectively, the data indicate that Nuc01 appears to exhibit dual functional characteristics: acting as a triple reuptake inhibitor potentially applicable for depression treatment and as an LSD1 inhibitor demonstrating anticancer potential.

## 1. Introduction

The global population of cancer survivors is on the rise, with projections indicating 1.95 million new cancer cases and 600,000 cancer-related deaths in the USA alone in 2023 [1]. These survivors typically face long-term physical and mental health challenges after completing their cancer treatments. Mental health issues, particularly depression, can have far-reaching negative impacts, including prolonged hospital stays, poor adherence to cancer therapies, reduced quality of life [2], heightened suicide risk [3,4,5], and increased psychological strain on the families of survivors [6,7]. Moreover, depression can disrupt immunological, endocrinological, and neurological functions, potentially exacerbating vulnerability to illness and increasing mortality rates [2]. There is also a distinct association between depressive symptoms and high mortality risks in cancer survivors [8], with severe depression emerging as an independent risk factor for death, separate from other medical variables [9,10]. Research has shown that depressive symptoms in cancer survivors are associated with high mortality rates and poor prognoses, suggesting that depression may play a role in disease progression [11,12].

Currently, second-generation antidepressants, particularly serotonin–norepinephrine reuptake inhibitors (SNRIs), such as venlafaxine, have demonstrated significant efficacy in reducing depressive symptoms. These medications have also been shown to help manage mood swings and hot flashes [13,14]. A systematic review and meta-analysis have confirmed that venlafaxine is significantly more effective in reducing hot flashes compared to placebo [15].

**Figure 1:**
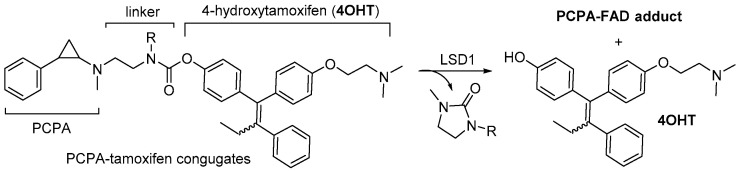
Prodrug activation mechanism for releasing the small-molecule drug in the PCPA–tamoxifen conjugates designed by Suzuki et al. [16].

The flavin adenine dinucleotide (FAD)-dependent lysine-specific demethylase 1 (LSD1) has become a focal point in cancer research as both a biomarker and a molecular target for cancer therapy. LSD1 primarily demethylates mono- and dimethylated lysine 4 of histone 3 (H3K4me1/me2) [16,17]. Overexpression or dysregulation of LSD1 has been observed in various cancers, including gastric cancer, small cell lung cancer (SCLC), breast cancer [18,19], neuroblastoma [20], acute myeloid leukemia (AML), retinoblastoma [21], and prostate cancer [22,23,24]. Furthermore, the targeted recruitment of LSD1 to promoter regions is linked to cancer cell proliferation [16]. To date, several LSD1 inhibitors have been identified [25,26,27], with most being based on *trans*-2-phenylcyclopropylamine (PCPA). PCPA inhibits LSD1 through a series of reactions, including a single-electron transfer, radical opening of the cyclopropyl ring, and the formation of a covalent bond with FAD [28,29]. During LSD1 inactivation, the imine intermediate is hydrolyzed, resulting in the release of the nitrogen atom from PCPA in the form of ammonia [16,17]. Based on this mechanism, in 2016, Suzuki et al. introduced PCPA–tamoxifen conjugates as novel small-molecule prodrugs that irreversibly block LSD1 by forming PCPA–FAD adducts while releasing the active drug 4OHT in an LSD1 enzyme-dependent manner (Figure 1). These findings highlight PCPA–drug conjugates (PDCs) as a promising strategy for enabling targeted drug activation in cancer cells [16,17].

Identifying the most effective and feasible interventions for treating depression in patients with advanced cancer remains a significant challenge in clinical oncology [30]. To address this, we developed a novel prodrug that targets cancer cells, while improving depressive symptoms. Nuc01 may possess dual functions: it appears to act as a novel triple reuptake inhibitor, potentially alleviating depressive symptoms in initial studies, and functions as an LSD1 inhibitor, exhibiting preliminary anticancer properties.

## 2. Materials and Methods

### 2.1. Chemicals and Reagents

Desmethylvenlafaxine (ODV) was obtained from Chengdu Beite Pharmaceutical Co., Ltd. (Chengdu, China). All other chemicals were sourced from Sigma–Aldrich (Shanghai, China) unless otherwise specified.

The synthesis of Nuc01 was carried out as follows: under an inert atmosphere of argon, pyridine (0.50 mL, 6.0 mmol) and p-nitrophenyl chloroformate (806.0 mg, 4.0 mmol) were added to a solution of desvenlafaxine (527.0 mg, 2.0 mmol) in dichloromethane (DCM, 20 mL) at 0 °C. The mixture was then warmed to room temperature and stirred for 5 h. The reaction mixture was subsequently diluted with DCM (60 mL), washed with water (20 mL), saturated sodium bicarbonate solution (20 mL), and brine (20 mL), and dried over sodium sulfate (Na_2_SO_4_). After filtration and concentration under reduced pressure, the activated carbonate of desvenlafaxine was obtained in the form of a brown oil.

Subsequently, a solution of the activated carbonate of desvenlafaxine in dry DCM (25 mL) was added to a suspension of N^1^,N^2^-dimethyl-N^1^-(2-phenylcyclopropyl)ethane-1,2-diamine (408.6 mg, 2.0 mmol, known compound [16]) in triethylamine (1.0 mL, 7.2 mmol) at 0 °C. This solution was then removed from the cooling bath, and the mixture was stirred at room temperature for 24 h. The reaction mixture was diluted with DCM (50 mL), washed with water (15 mL) and brine (15 mL), and the organic layer was dried over Na_2_SO_4_. After filtration and concentration under reduced pressure, the product was purified via flash column chromatography on silica gel (MeOH/CHCl_3_: *v*/*v* = 1/20) to yield Nuc01 (479.5 mg, 49% yield for two steps) as a pale yellow, amorphous solid. The structure of Nuc01 was confirmed by ^1^H nuclear magnetic resonance (600 MHz, CDCl_3_), ^13^C nuclear magnetic resonance (150 MHz, CDCl_3_), and high-resolution mass spectrometry (see Figures S1–S3 in ESI). ^1^H NMR (600 MHz, CDCl_3_) δ 7.26–7.22 (m, 2H), 7.15 (t, *J* = 6.6 Hz, 1H), 7.12–7.08 (m, 2H), 7.08–7.02 (m, 3H), 6.98 (d, *J* = 8.4 Hz, 1H), 3.59–3.43 (m, 2H), 3.28 (t, *J* = 12.6 Hz, 1H), 3.10–2.97 (m, 4H), 2.86–2.77 (m, 2H), 2.43 (s, 3H), 2.32 (s, 6H), 2.32–2.29 (m, 1H), 2.00–1.95 (m, 2H), 1.76–1.69 (m, 2H), 1.67–1.62 (m, 1H), 1.53 (dd, *J* = 24, 10.8 Hz, 3H), 1.37–1.35 (m, 1H), 1.33–1.28 (m, 1H), 1.12–1.08 (m, 1H), 1.02–0.95 (m, 2H), 0.90–0.84 (m, 1H). ^13^C NMR (150 MHz, CDCl_3_) δ 154.9, 150.3, 142.1, 142.0, 137.6, 130.1, 128.4, 126.1, 125.8, 125.8, 121.3, 121.2, 74.4, 61.2, 55.7, 54.9, 52.1, 49.5, 49.4, 47.5, 47.4, 45.6, 42.5, 42.4, 38.2, 35.4, 35.2, 31.3, 26.1, 25.9, 25.7, 21.7, 21.4, 17.8, 17.7. HRMS calculated for C_30_H_43_N_3_O_3_ [M + H]^+^ 494.3377; found 494.3373.

### 2.2. Molecular Docking

Molecular docking was performed using AutoDock 4.2 with AutoDockTools (ADT) 1.5.7. The crystal structure of LSD1-CoREST in complex with (+)-*trans*-2-phenylcyclopropyl-1-amine (PDB code: 2XAH) was used to construct the molecular docking model for predicting the binding mode of Nuc01. The protein structure was prepared by removing all water molecules and non-relevant ligands, adding polar hydrogen atoms, and assigning Gasteiger charges using ADT. The docking grid was centered on the co-crystallized ligand (+)-*trans*-2-phenylcyclopropyl-1-amine, with grid box dimensions large enough to cover the active site region. The 3D structure of Nuc01 was generated and energy-minimized using Open Babel (Version 2.4.1) and converted to the required PDBQT format using ADT. All rotatable bonds in the ligand were allowed to rotate during docking. Docking simulations were carried out using the Lamarckian Genetic Algorithm (LGA) with default parameters: 100 runs, a population size of 150, and 2.5 million energy evaluations. The best binding pose was selected based on the lowest binding energy and visual inspection of the interaction mode.

### 2.3. In Vitro LSD1 Inhibitor Experiment

The LSD1 enzyme (residues 172–852) was used in conjunction with synthesized Nuc01 and the LSD1 inhibitor tranylcypromine (TCP; positive control). A biotinylated peptide substrate was diluted in assay buffer just before use. Initially, 10 µL of 2× inhibitor (or assay buffer as control), Nuc01 at various concentrations (starting at 100 µM, serially diluted at a 1:1 ratio), and 5 nm LSD1 enzyme were added to a 96-well plate and incubated at room temperature for 30 min. Subsequently, 5 µL of 4× biotinylated histone H3K4me1 peptide substrate was added to each well and incubated for 1 h at room temperature. A stop solution containing 300 µM TCP in 1× LANCE detection buffer (Perkin Elmer) was added to the wells and incubated for 5 min at room temperature. A detection mix, consisting of 2 nm Eu-labeled antibody and 50 nm ULight-Streptavidin (Perkin Elmer, Shelton, CT, USA) in 1× LANCE detection buffer, was prepared and added to the reaction mixture. After incubating for 1 h at room temperature, the plate was read using the Pherastar Reader in TR-FRET mode (excitation at 337 nm, emission at 665 nm, and 620 nm).

### 2.4. LSD1 Inhibitor in MDA-MB-231 Cells

Human triple-negative breast cancer cells (MDA-MB-231) were purchased from the Shanghai Cell Bank of Chinese Academy of Science (Shanghai, China). Human triple-negative breast cancer cells (MDA-MB-231) were cultured in high-glucose Dulbecco’s modified eagle medium. Log-phase cells were transferred to a 96-well plate. Each well received 100 µL of the cell suspension, which was mixed evenly and incubated in a CO_2_ incubator for 24 h. A pre-prepared 20 mm compound was diluted in serum-free culture medium to a concentration of 100 µM, then serially diluted to six different concentrations, with DMSO as the negative control. Each gradient concentration was approximately 360 µL to ensure three replicates for each compound at every concentration. The old culture medium was discarded, and serum-free medium containing the selected compound was added, followed by incubation in a CO_2_ incubator for an additional 72 h. After 72 h, 10 µL of 3-(4,5-dimethylthiazol-2-yl)-2,5-diphenyltetrazolium bromide (MTT) (5 mg/mL) was added to each well in the absence of light, followed by incubation at 37 °C for 4 h. The supernatant was discarded, and 100 µL of DMSO was added to each well. The plate was cleaned before reading the absorbance at 490 nm on a microplate reader. Cell viability was calculated using the following formula: survival rate = (OD treatment group/OD control group) × 100%, with data analyzed using GraphPad Prism 7 (Version 7.00, GraphPad Software Inc., La Jolla, CA, USA).

### 2.5. Tail Suspension Test (TST) in Mice

Male C57BL/6 mice, weighing between 18 and 22 g, were acquired from Shanxi Medical University in Taiyuan, China. All mouse experiments were performed in accordance with the ARRIVE guidelines and were approved by the Biological and Medical Ethics Committee of North University of China (202412-120). The mice were housed in a controlled environment with a temperature maintained at 22.0 ± 2.0 °C and relative humidity kept at 50 ± 10%, under a 12 h light/dark cycle. The mice had ad libitum access to food and water. All experimental procedures were conducted in strict accordance with the National Institute of Health Guide for the Care and Use of Laboratory Animals (NIH Publications No. 80–23, revised in 1996).

TST in mice has been shown to be sensitive to a broad range of antidepressants [20]. In total, 50 C57BL/6 mice were randomly assigned to five groups (*n* = 10/group) and were intragastrically administered with ODV (15 mg/kg), Nuc01 (7.5, 15, and 30 mg/kg), or vehicle 60 min before testing. Mice were individually suspended by their tails approximately 35 cm above a tabletop using adhesive tape placed approximately 1 cm from the tip of the tail. A mouse was considered immobile only when hanging passively and completely motionless. The test session was recorded for 6 min, and the immobility time was determined by an observer who was blinded to the treatment conditions.

## 3. Results

### 3.1. Design Concept of Synergistic Antidepressant and Anticancer Therapy Through the Combination of Two Drugs

**Figure 2:**
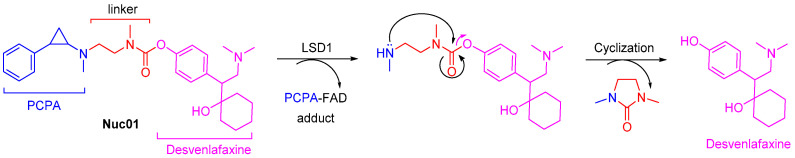
Design concept of the PCPA–desvenlafaxine conjugate (Nuc01) as a small molecule-based drug delivery molecule.

Guided by the insights from a study by Ota et al. [16], PCPA, a first-generation antidepressant, was strategically designed as a warhead targeting LSD1 (Figure 2). Desvenlafaxine, a 5-HT/NA dual reuptake inhibitor and the O-demethylated metabolite of venlafaxine (approved in 1993), was then conjugated to PCPA. This design leverages the ability of PCPA to react with the FAD cofactor in the LSD1 protein, thereby blocking the enzymatic activity of LSD1. Concurrently, it releases desmethylvenlafaxine in vivo to treat depression, ultimately achieving a synergistic therapeutic effect (Figure 2). Following the design phase, Nuc01 was synthesized using established organic chemistry techniques.

### 3.2. Analysis of the Binding Mode Between Nuc01 and Its Target LSD1

**Figure 3:**
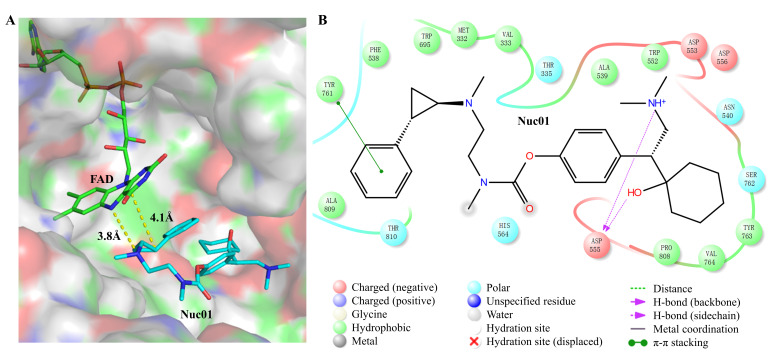
Predicted binding mode of Nuc01. The predicted binding mode of Nuc01. (**A**) Nuc01 located in the PCPA pocket of LSD1. The LSD1 protein is displayed in surface representation, while the small molecule Nuc01 is shown as cyan sticks. The cofactor FAD, which interacts with Nuc01, is represented as green sticks. The distances between Nuc01 and FAD are indicated by dashed lines. (**B**) The detailed interactions between Nuc01 and LSD1. All residues of the LSD1 protein that interact with Nuc01 are shown in the figure. Green-colored residues indicate hydrophobic interactions, cyan residues represent polar interactions, and purple arrows denote hydrogen bonds. Green dashed lines indicate π–π stacking interactions, while dark red residues represent negatively charged amino acids.

**Figure 4:**
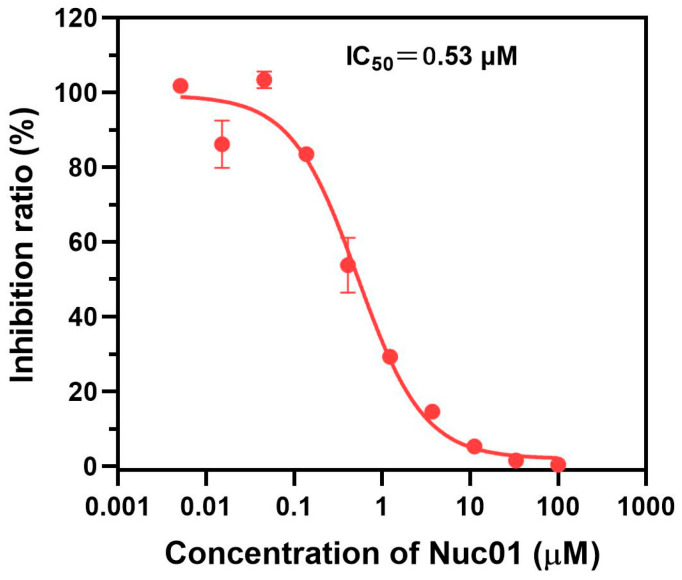
IC_50_ curve of Nuc01 against LSD1 at the enzymatic level.

The binding mode between Nuc01 and LSD1 was probed by molecular docking. Molecular docking results reveal that our designed PCPA–desvenlafaxine conjugate, Nuc01, effectively occupies the PCPA binding site within the LSD1 protein (Figure 3A). It establishes multiple interactions, including hydrophobic, hydrogen bonding, and π–π interactions (Figure 3B). These interactions stabilize Nuc01 firmly within the binding pocket, with a binding energy of −6.508 kcal/mol. Specifically, Nuc01 engages in strong hydrophobic interactions with residues Y761, F538, W695, M332, V333, A809, A539, W552, P808, V764, and W763. Additionally, D555 forms a hydrogen bond with Nuc01, while Y761 contributes a π–π interaction. Moreover, the PCPA moiety of Nuc01 is positioned in close proximity to the FAD region (Figure 3A), facilitating the reaction between PCPA and FAD to form the PCPA–FAD conjugate. This observation aligns with our activity assay results, which demonstrate that Nuc01 exhibits potent inhibitory activity against LSD1, with an IC_50_ of 530 nm (Figure 4).

### 3.3. Anti-Proliferative Effects of Nuc01 on MDA-MB-231 Cells

**Figure 5:**
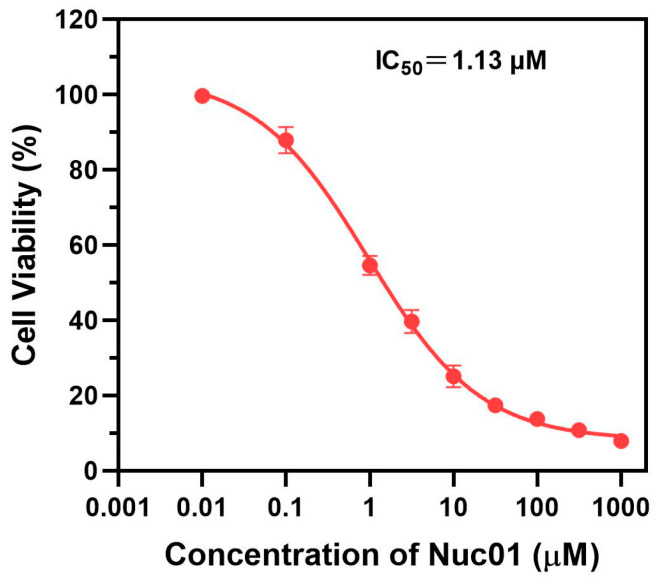
In vitro cytotoxicity assay of various Nuc01-containing drugs on MDA-MB-231 cells.

The anti-proliferative effects of Nuc01 were evaluated using an MTT assay on the human triple-negative breast cancer cell line MDA-MB-231. The results (Figure 5) demonstrate that Nuc01 significantly inhibits the proliferation of MDA-MB-231 cells, with an IC_50_ of 1130 nm.

### 3.4. Effect of Nuc01 on the Tail Suspension Test (TST) in Mouse

**Figure 6:**
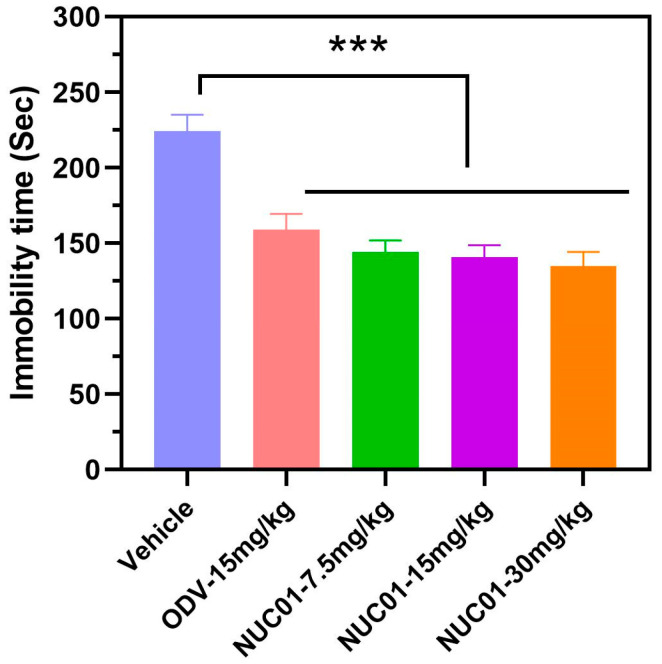
Antidepressant therapeutic effect of different treatments: vehicle, ODV, and various doses of Nuc01 (7.5, 15, and 30 mg/kg) on the TST in C57BL/6 mice. All results are expressed as the mean ± SD from ten independent experiments (*n* = 10), and the significance levels are *** *p* < 0.001, analyzed by a one-way ANOVA with a Tukey’s test.

After confirming that Nuc01 exhibits strong inhibitory activity against both the LSD1 enzyme and tumor cell proliferation, we subsequently evaluated its antidepressant activity using the TST in mouse. As shown in Figure 6, a one-way ANOVA with a Tukey’s test revealed significant differences in immobility time among the groups in the TST. Post hoc analysis showed that Nuc01 (7.5, 15, and 30 mg/kg) significantly reduced immobility time compared to that in the vehicle control group (*p* < 0.001). Notably, Nuc01 (15 mg/kg) exhibited greater potency than ODV at the same dose (31% vs. 38%).

## 4. Discussion

In this study, we comprehensively characterized the in vitro and in vivo pharmacological profile of Nuc01, which is a novel compound with a unique structure. Our findings provide robust evidence that Nuc01 may serve dual functions. Nuc01, acting as a prodrug of venlafaxine, was observed to alleviate symptoms of depression with promising efficacy. Furthermore, its activity as an LSD1 inhibitor was associated with anticancer effects in the studied models.

Recent preclinical studies and clinical trials have indicated that triple reuptake inhibitors (TRIs) may offer faster onset of action, greater efficacy, and fewer side effects than traditional antidepressants [31,32,33]. Guided by the insights from a study by Ota et al. [16], Nuc01 releases desmethylvenlafaxine in vivo to treat depression, ultimately achieving a synergistic therapeutic effect. Therefore, Nuc01, a prodrug of venlafaxine, is a novel SNRI with significant antidepressant properties. To identify TRIs with favorable in vivo profiles, we used a behavioral screening approach, focusing on tests predictive of antidepressant activity in humans, such as the TST in mice [34]. In the present study, Nuc01 significantly decreased immobility time in the TST at doses of 7.5, 15, and 30 mg/kg (Figure 6). Moreover, Nuc01 exhibited greater potency than ODV at the same doses. As false positives can occur in the TST for compounds that stimulate spontaneous locomotor activity [35], we assessed spontaneous locomotor activity to rule out this potential confounding effect. The results showed that Nuc01 did not increase spontaneous locomotor activity in mice at doses of 7.5, 15, and 30 mg/kg.

Overall, compared to venlafaxine, a commonly prescribed SNRI, Nuc01 displayed higher antidepressant efficacy in vivo. We believe that such a “broad-spectrum” antidepressant could represent a significant breakthrough in the treatment of depression in cancer survivors.

Histone lysine-specific demethylase 1 (LSD1) has emerged as an important and promising anticancer target since its initial identification by Shi et al. in 2004 [36]. LSD1 specifically demethylates lysine residues of histone H3K4me1/2 and is overexpressed in various cancers. Abrogation of LSD1 has been shown to inhibit cancer cell proliferation, invasion, and migration. Over the past decade, numerous LSD1 inhibitors have been developed [35,37,38,39,40,41,42]. To date, six PCPA-based LSD1 inhibitors, including TCP, ORY-1001 [43], GSK-2879552 [31,44], INCB059872 [32,33], IMG-7289, and ORY-2001, have reached the clinical trial stage, either alone or in combination with other therapies, for the treatment of cancers and neurodegenerative disorders [34,37,45]. PDCs have the potential to selectively target cancer cells with high LSD1 expression [16,17]. In our in vitro studies, Nuc01 displayed potent inhibitory activity against the LSD1 enzyme, with an IC_50_ of 0.53 µM (Figure 4), and significantly inhibited the proliferation of the human cancer cell line MDA-MB-231, with an IC_50_ of 1.13 µM (Figure 5). As an LSD1 inhibitor, Nuc01 showed remarkable anticancer efficacy. Given that Nuc01 is structurally derived from the PCPA (tranylcypromine) scaffold, which is known to induce irreversible, mechanism-based inhibition of LSD1 through covalent adduct formation with the FAD cofactor, we anticipate that Nuc01 likely exerts its inhibitory effect through a similar irreversible mechanism. Although we have not yet performed detailed kinetic assays (e.g., time-dependent IC_50_ shift or dialysis recovery) to confirm the covalent nature of inhibition, the structural resemblance to known irreversible LSD1 inhibitors (e.g., ORY-1001 [43] and GSK2879552 [31,44]) and the durable suppression of enzymatic activity observed in our assays support this hypothesis. Future studies including time-dependent inhibition profiles and mass spectrometry-based covalent binding analysis are warranted to validate this assumption.

Furthermore, endocrine therapy is the first-line treatment for patients with hormone receptor-positive breast cancer, according to current guidelines [46]. Approximately 40–50% of patients with breast cancer undergoing chemotherapy experience premature menopause symptoms, including hot flashes. Some endocrine therapies, such as tamoxifen and aromatase inhibitors, are associated with inducing or exacerbating hot flashes. These symptoms can be debilitating and can significantly impair daily functioning. Therefore, various therapeutic options have been explored to manage this adverse effect. Venlafaxine has been shown to be efficacious in improving hot flashes [15,47]. Barton investigated the etiology and treatment of hot flashes and reported that venlafaxine reduced hot flash symptoms by approximately 60% [48]. The Oncology Practice Development Committee (CEPO) recommends the use of venlafaxine, citalopram, clonidine, gabapentin, and pregabalin for treating hot flashes in patients with breast cancer receiving tamoxifen [41]. As a prodrug of venlafaxine, Nuc01 may similarly reduce hot flashes, making it a potentially more suitable option for treating breast cancer.

Based on the substantial evidence supporting the efficacy of antidepressants and anticancer agents, our results suggest that the antidepressant activity of Nuc01 is likely to enhance its anticancer reactivity. Moreover, Nuc01 may be particularly well-suited for the treatment of breast cancer.

## 5. Conclusions

In conclusion, Nuc01, a novel prodrug combining venlafaxine and the LSD1 inhibitor PCPA, demonstrated dual therapeutic potential. In vitro, Nuc01 showed strong binding affinity to LSD1 and effectively inhibited the proliferation of MDA-MB-231 cancer cells. In vivo, Nuc01 outperformed desvenlafaxine in reducing depression-like behavior (e.g., immobility time in mice tail suspension tests) in a dose-dependent manner.

## Data Availability

The diverse data generated and analyzed during this work are available from the corresponding authors on request.

## Supplementary Materials

The following supporting information can be downloaded at https://www.mdpi.com/article/10.3390/cimb47080587/s1.

## Abbreviations

The following abbreviations are used in this manuscript:

SNRIs	Serotonin and norepinephrine reuptake inhibitors
LSD1	Lysine-specific demethylase 1
PCPA	*Trans*-2-phenylcyclopropylamine
FAD	Flavin adenine dinucleotide
ODV	Desmethylvenlafaxine
TST	Tail suspension test
