# Supplementary material for: Effect of a Novel Antidepressant and Anticancer Nuc01 on Depression in Cancer Survivors
Source: Curr Issues Mol Biol. 2025 Jul 24;47(8):587. doi: 10.3390/cimb47080587 (PMC12384447; doi:10.3390/cimb47080587)
Supplement: Supplementary file 1 [file cimb-47-00587-s001.zip › cimb-3729650-supplementary.pdf]

Supporting Information

Effect of a novel antidepressant and anticancer Nuc01 on depression in cancer survivors

Changchun Yuan[1, 2, *], Xudong Shi[1], Zhiqiang Wang[1,*], Yuqiang Li[1], Wenbing Ma[1] and Kai Fu[1, 2]

[1] School of Chemistry and Chemical Engineering, North University of China, Taiyuan 030051

[2] Dezhou Industrial Technology Research Institute, North University of China, Dezhou 253000

Spectra for Compound Nuc01

Figure S1. ^{1}H NMR spectra of compound **Nuc01**

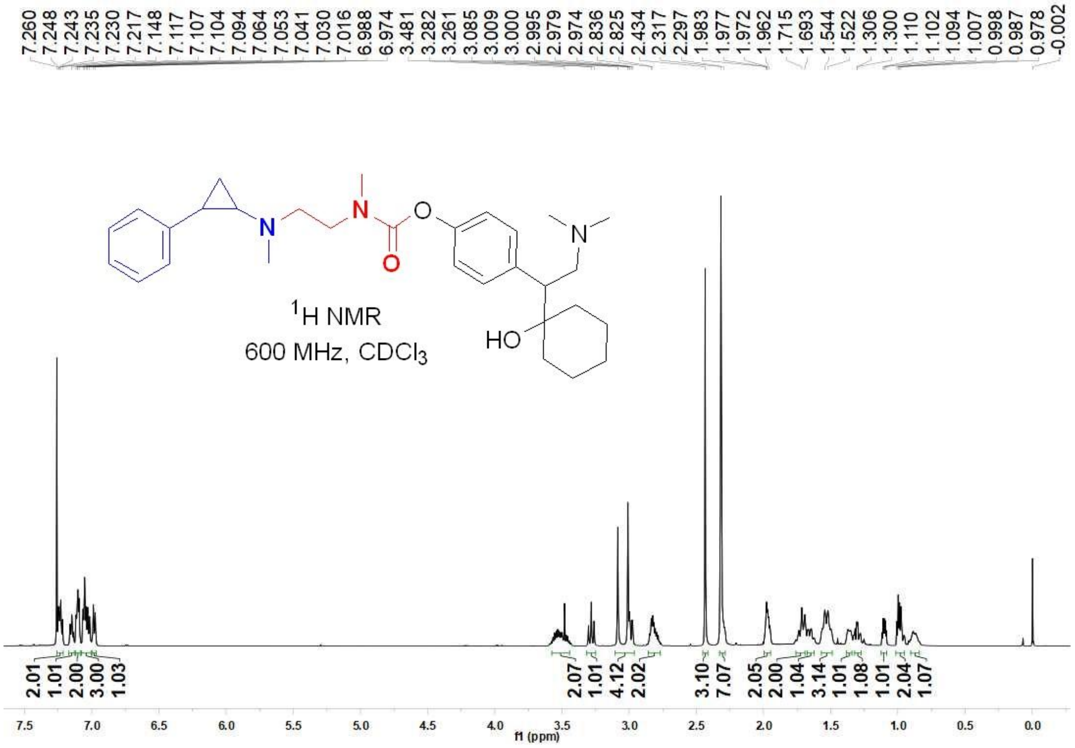

Figure S2. ^{13}C NMR spectra of compound **Nuc01**

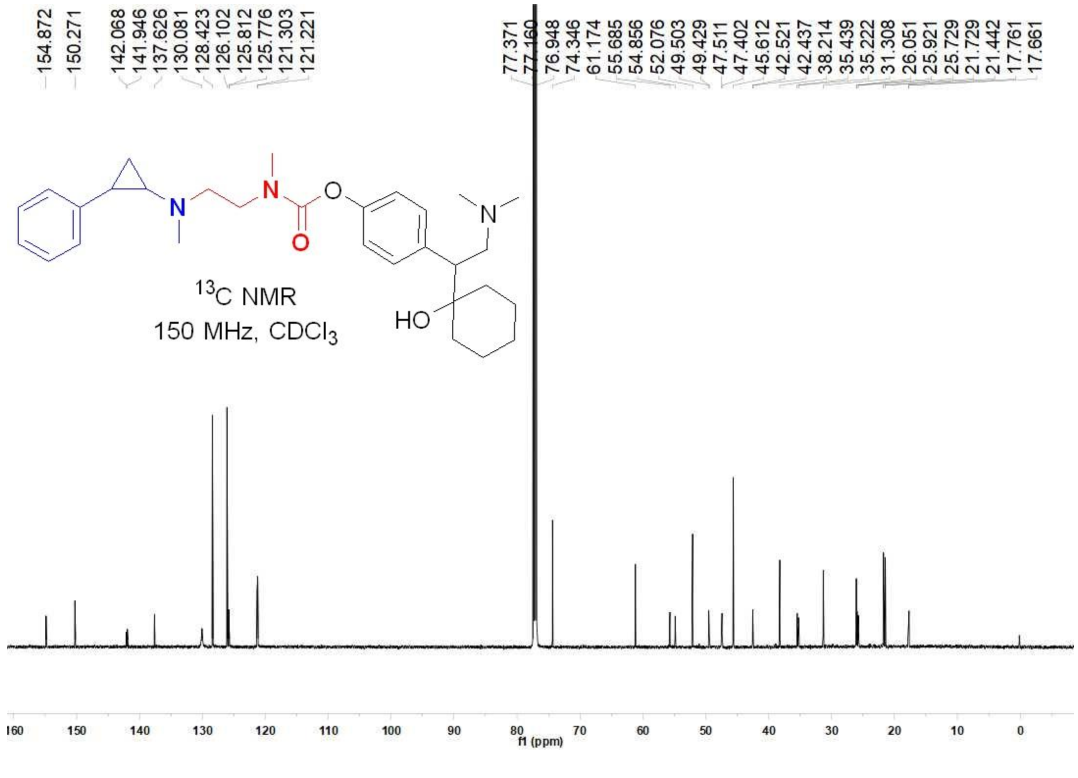

Figure S3. The (+)-HR-ESIMS spectrum of **Nuc01**.

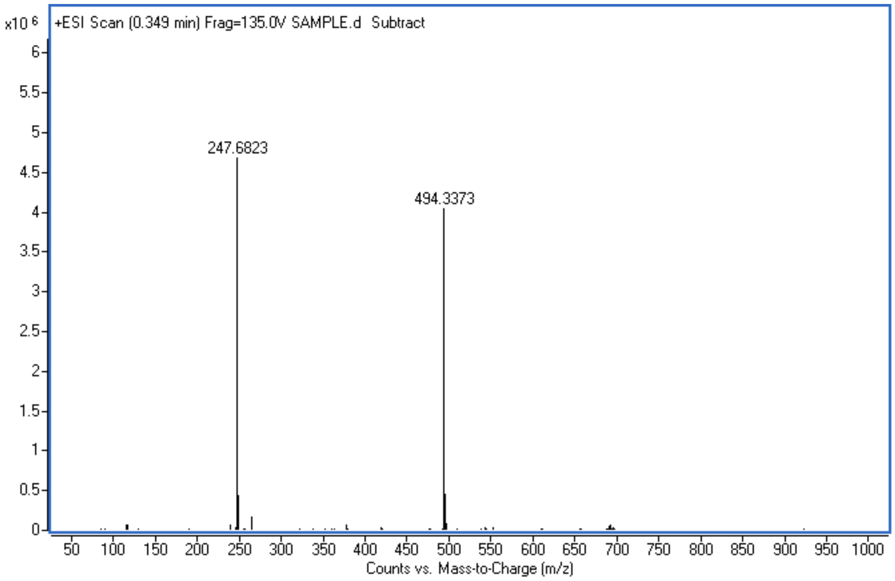

HRMS calcd for $C_{30}H_{43}N_3O_3$ $[M+H]^+$ 494.3377, found 494.3373.
